# Human Adipose Tissue Macrophages Are Enhanced but Changed to an Anti-Inflammatory Profile in Obesity

**DOI:** 10.1155/2014/309548

**Published:** 2014-03-11

**Authors:** Karen Fjeldborg, Steen B. Pedersen, Holger J. Møller, Tore Christiansen, Marianne Bennetzen, Bjørn Richelsen

**Affiliations:** ^1^Department of Medicine and Endocrinology, MEA, Aarhus University Hospital, 8000 Aarhus, Denmark; ^2^Department of Clinical Biochemistry, Aarhus University Hospital, 8000 Aarhus, Denmark

## Abstract

*Objective*. Adipose tissue (AT) macrophages are increased in obesity and associated with low grade inflammation. We aimed to characterize the phenotype of AT macrophages in humans in relation to obesity and insulin resistance.* Design*. Gene-expression levels of general macrophage markers (CD68 and CD14), proinflammatory markers/M1 (TNF-**α**, MCP-1, and IL-6), and anti-inflammatory markers/M2 (CD163, CD206, and IL-10) were determined by RT-PCR in subcutaneous AT samples from lean and obese subjects. Insulin resistance was determined by HOMA-IR.* Results*. All the macrophage markers were elevated in the AT from obese compared to lean subjects (*P* < 0.001). To determine the phenotype of the macrophages the level of CD14 was used to adjust the total number of macrophages. The relative expression of CD163 and IL-10 was elevated, and TNF-**α** and IL-6 were reduced in AT from obese subjects (all *P* < 0.05). In a multivariate regression analysis CD163 was the only macrophage marker significantly associated with HOMA-IR (**β**: 0.57; *P* < 0.05). Conclusion. Obesity is associated with elevated numbers of macrophages in the AT. Unexpectedly, the macrophages change phenotype by obesity, with a preponderance of M2 and a decrement of M1 markers in AT from obese subjects. Moreover, CD163 was the only macrophage marker associated with HOMA-IR after multiple adjustments.

## 1. Introduction

In obese subjects there are an increased number of macrophages in the adipose tissue (AT), which produce several cytokines that contribute to local AT inflammation and to systemic low grade inflammation [1–3]. It is believed that adipocyte hypertrophy and local hypoxia due to adipocyte expansion are two important contributing factors to the increased accumulation of macrophages in AT in the obese state [4, 5]. The adipose tissue macrophages (ATMs) of obese subjects are often located in “crown like structures” (CLS) surrounding dead adipocytes [6] and they are also found in elevated numbers in fibrotic areas in the AT [7]. ATMs may have a scavenger function in response to necrotic adipocytes, but the role in regard to fibrosis is unclear.

Macrophage phenotypes are often divided into pro- (M1) or anti-inflammatory (M2) subpopulations. The M1 or classical activated macrophages are induced by, for example, LPS and TNF-*α* and produce proinflammatory cytokines. The M2 or alternative activated macrophages are induced by, for example, glucocorticoids, IL-4, and IL-10 and produce anti-inflammatory cytokines [5, 8–10]. An intermediate phenotype has also been described, which resembles anti-inflammatory M2 markers but at the same time secretes large amounts of proinflammatory cytokines [11, 12]. In obese mice an increased number of macrophages in the AT are observed which is similar to the human situation. In rodent models diet-induced obesity generally leads to a shift in the phenotype in ATMs from a M2-polarized state in lean animals to a M1-polarized state in obese animals [13, 14]. In humans the results concerning changes in polarization in obese subjects are less clear. Some studies have shown that the levels of proinflammatory markers in subcutaneous ATMs are elevated in obese subjects compared with lean subjects [3, 15]. And it has been shown that weight loss induced by either very low energy diet (VLED) or gastric bypass induces an increased level of anti-inflammatory macrophage markers and reduced level of proinflammatory macrophage markers in subcutaneous AT [3, 15, 16]. However, one study has shown that subcutaneous ATMs change to a more anti-inflammatory phenotype by obesity [7].

Chronic low grade inflammation in obesity is associated with insulin resistance, which predispose to the development of type 2 diabetes. Several studies have described an association between ATMs and insulin resistance [2] and a recent study has described a positive association between proinflammatory macrophages in the AT and systemic insulin resistance measured by HOMA-IR [17].

With the present study we wanted to examine whether the phenotype (polarization) of ATMs is changed in the obese state in humans. As proinflammatory markers we used the gene-expression level of monocyte chemoattractant protein-1 (MCP-1), tumor necrotic factor-*α* (TNF-*α*), and interleukin 6 (IL-6). MCP-1 is involved in the recruitment of macrophages and we have earlier found that mRNA MCP-1 levels in human AT samples correlate with measures of adiposity [18]. Both TNF-*α* and IL-6 are known proinflammatory mediators, which have been used as proinflammatory markers in other studies [19, 20]. As anti-inflammatory markers we used the gene expression of CD163, CD206, and interleukin 10 (IL-10), all of them frequently used as anti-inflammatory markers in the literature [3, 11, 19, 20]. CD206 is a mannose receptor and CD163 is a scavenger receptor involved in clearance of haptoglobin-hemoglobin complexes, from ruptured red blood cells [21, 22]. Soluble CD163 (sCD163) is the extracellular part of CD163 and it is found to be shed of the receptor during proinflammatory conditions and in obesity [23–26].

We hypothesized that there is a shift in phenotype towards an increased level of proinflammatory macrophage markers in AT from obese subjects compared with AT from lean subjects as generally found in rodent models. Furthermore, we wanted to study the relationship between insulin resistance at the whole body level measured by HOMA-IR and pro- and anti-inflammatory macrophage markers in AT.

## 2. Material and Methods

### 2.1. Subjects

Fat biopsies were obtained from the subcutaneous abdominal AT and originated from two previously performed studies. Only baseline fat biopsies were used in the present study. Study 1: AT samples were obtained from 21 lean, healthy controls and 21 obese and otherwise healthy subjects who participated in a weight loss program with VLED for 12 weeks, as previously described [27]. Study 2: AT samples were obtained from 14 lean, healthy controls and 36 obese and healthy subjects, who participated in a 12-week weight loss intervention program with exercise alone, VLED, or a combination of exercise and VLED [28]. Both studies took place at the research laboratory at Aarhus University Hospital. All subjects were recruited via advertisements in local newspapers. None of the subjects had type 2 diabetes or took medicine that could affect inflammation or adipose tissue metabolism. Inclusion and exclusion criteria are previously described [27, 28]. The subjects were all Caucasian and had a sedentary life style. The study was approved by the local ethics committee in the county of Aarhus and followed the principles of the Declaration of Helsinki.

### 2.2. Anthropometrics and Blood Samples

Anthropometrics and fasting blood samples were collected after an overnight fast. All participants were asked not to engage in excessive physical exercise or alcohol intake the day before or in the morning of clinical investigations. Venous blood samples were collected and serum frozen at −80°C. The homeostasis model assessment insulin resistance index (HOMA-IR) was calculated using the formula of serum fasting insulin (*μ*U/mL) ∗ fasting glucose (mmol/L)/22.5 [29, 30]. Soluble CD163 was quantified in serum samples using an in-house enzyme-linked immunosorbent assay, as previously described [31]. IL-6 was measured with a high sensitivity ELISA (R&D Systems, USA) and MCP-1 was measured with a human ELISA DuoSet (R&D systems). sCD163, MCP-1, and IL-6 were measured and described in a previous study [23].

### 2.3. AT Biopsy

Subcutaneous abdominal adipose tissue was obtained from 57 obese subjects and 35 lean subjects in total. The biopsies were obtained by liposuction from the subcutaneous abdominal adipose depot in local anesthesia under sterile conditions. Immediately after removal, the adipose tissue sample was washed in isotonic NaCl, snap-frozen in liquid nitrogen, and kept at −80°C until RNA extraction. The procedures are described in detail elsewhere [27, 28].

### 2.4. mRNA Isolation and RT-PCR Analysis

RNA was isolated using TRIzol reagent (GIBCO-BRL Life Technologies, Roskilde, Denmark), and cDNA was synthesized using random hexamer primers using the Verso cDNA Kit (Applied Biosystems). All analyses were performed simultaneously and the mRNA levels of the target genes were expressed relative to the house-keeping gene low-density lipoprotein receptor related protein-10 (LRP10). The PCR reactions were performed in duplicate using the KAPA SYBR FAST qPCR Kit (Kapa Biosystems, Inc., Woburn, MA, USA) in a LightCycler 480 (Roche Applied Science) using the following protocol: one step at 95°C for 3 min, then 95°C for 10 s, 60°C for 20 s, and 72°C for 10 s. The increase in fluorescence was measured in real time during the extension step. The relative gene expression was estimated using the default “Advanced Relative Quantification” mode of the software version LCS 480 1.5.0.39 (Roche Applied Science). The primers were designed using QuantPrime software [32]. The specificity was tested and amplification efficiency determined (between 1.9 and 2.0). Before analysis of target genes, the house-keeping gene was tested for stability and found to be stable comparing both groups and displaying comparable number of *C*_*T*_ cycles. The primer pairs are listed in Table 1.

### 2.5. Statistical Analysis

Descriptive statistics for anthropometrics and HOMA-IR are presented as mean ± SD. Baseline unpaired data were analyzed with an unpaired *t*-test or a Wilcoxon Mann-Whitney rank sum test for those variables, which were not normally distributed. To analyze the association between the cytokines, HOMA-IR, and the different macrophage markers we used a Spearman's correlation test. Multivariate linear regression was performed on a log scale to adjust the total number of macrophages and to find the macrophage marker with the closest association to HOMA-IR. The chosen significance level was a two-tailed *P* value of <0.05. The statistical software packet SPSS (SPSS, Chicago, IL, USA) was used for all calculations. Graphs were made in SigmaPlot.

## 3. Results

Characteristics of the lean and obese group are given in Table 2. Mean BMI of the obese subjects was 35.6 ± 3.8 kg/cm^2^ and the lean subjects 23.2 ± 1.8 kg/cm^2^. Age was between 18 and 49 years and was similar between the two groups. The level of HOMA-IR was significantly higher in the obese subjects than in the lean subjects (3.8 ± 1.8 versus 1.8 ± 0.8, *P* < 0.001). Furthermore, there was a significantly higher level of sCD163, MCP-1 (for both, *P* < 0.001), and IL-6 (*P* < 0.05) in blood samples from obese subjects compared with the lean subjects (Table 2).

### 3.1. Expression of Macrophage Markers in AT

**Figure 1:**
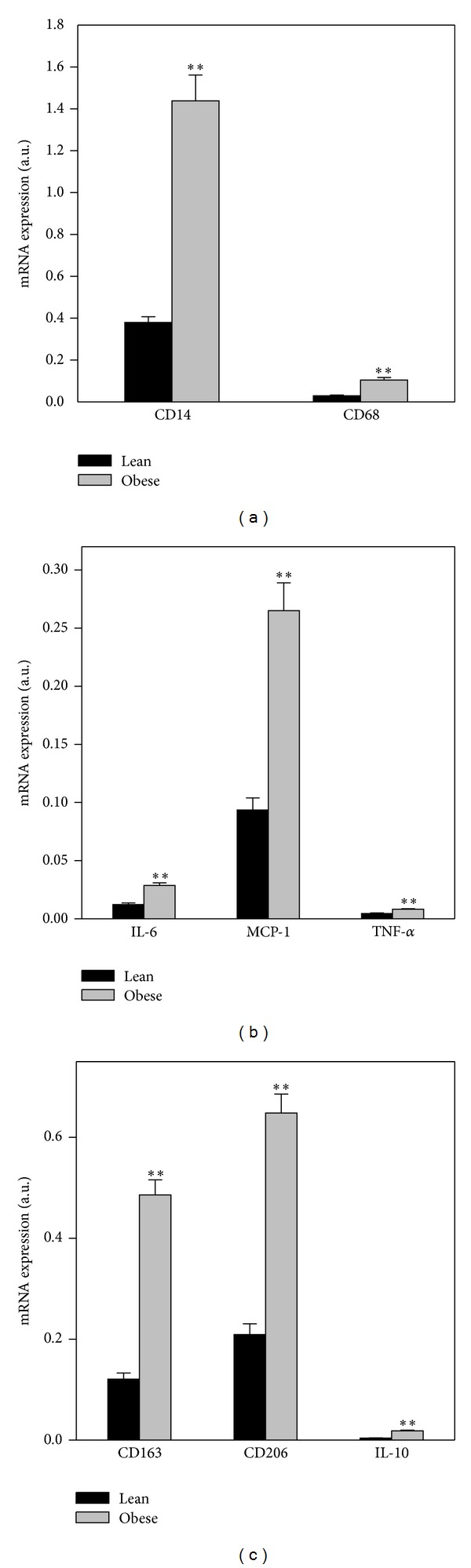
Difference in macrophage markers in AT between lean and obese subjects. Subcutaneous abdominal adipose tissue samples from lean (*n* = 35) and obese (*n* = 57) subjects. Gene-expression levels of different macrophage markers measured by real time PCR. (a) The general macrophage markers CD14 and CD68 in ATMs from lean and obese subjects. (b) Proinflammatory markers, IL-6, MCP-1, and TNF-*α*, in ATMs from lean and obese subjects. (c) Anti-inflammatory markers CD163, CD206, and IL-10 in ATMs from lean and obese subjects. Wilcoxon Mann-Whitney rank sum test. **P < 0.001.

The general macrophage markers CD68 and CD14 were taken as close correlates to the total number of macrophages, and both markers were significantly elevated in AT from obese subjects compared with lean subjects (*P* < 0.001) (Figure 1(a)). The levels of CD68 and CD14 were not significantly different between the genders and were not associated with age (data not shown). The gene expression of the proinflammatory markers IL-6, MCP-1, and TNF-*α* were all significantly elevated in the AT from obese subjects compared with lean subjects (for all, *P* < 0.001) (Figure 1(b)). Likewise there was a significantly higher level of the anti-inflammatory markers CD163, CD206, and IL-10 in the AT from obese subjects compared with AT from lean subjects (for all, *P* < 0.001) (Figure 1(c)). The gene expression of CD14 and CD68 were positively and significantly associated with the anti-inflammatory markers: CD163 (*r*: 0.76; *r*: 0.73), CD206 (*r*: 0.86; *r*: 0.78), and IL-10 (*r*: 0.73; *r*: 0.67), and with the proinflammatory markers: TNF-*α* (*r*: 0.45; *r*: 0.40), MCP-1 (*r*: 0.73; *r*: 0.74), and IL-6 (*r*: 0.56; *r*: 0.47)(for all, *P* < 0.001).

### 3.2. Differences in the Macrophage Phenotypes

**Figure 2:**
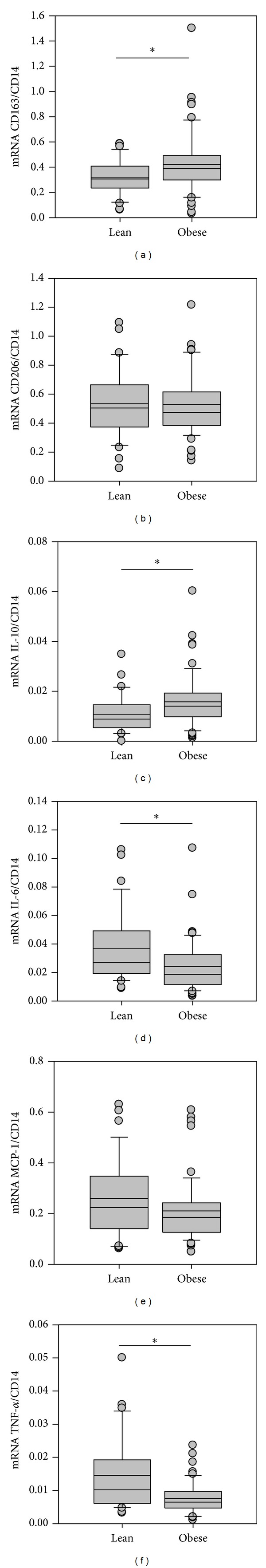
Polarization of anti- and proinflammatory macrophage markers in lean and obese subjects. Difference in phenotype of macrophage markers is expressed in subcutaneous abdominal adipose tissue samples from lean (*n* = 35) and obese (*n* = 57) subjects. Gene-expression levels of macrophage markers are presented relative to CD14, measured by RT-PCR. Lean versus obese analyzed by a Wilcoxon Mann-Whitney rank sum test. The ratio between the anti-inflammatory markers CD163, CD206, and IL-10 ((a)–(c)). The ratio between the proinflammatory markers IL-6, MCP-1, and TNF-*α* ((d)–(f)). *P < 0.05. Graph showing each outliner and mean value with a solid line.

To determine the changes in the polarization of the macrophages, the ratio of the different anti- and proinflammatory markers relative to CD14 was examined. As shown in Figures 2(a)–2(c) the level of the anti-inflammatory markers, CD163 and IL-10 adjusted for CD14, was significantly elevated in AT from obese compared to lean subjects (*P* < 0.05). The ratio of the proinflammatory markers, TNF-*α* and IL-6 adjusted for CD14, was significantly lower in AT from obese subjects compared to lean individuals (*P* < 0.05) (Figures 2(d)–2(f)). There was no difference in the level of CD14+ macrophages expressing CD206 and MCP-1 between lean and obese subjects. Using CD68 showed similar results as with CD14 (data not shown).

### 3.3. Association between Protein Levels and Gene-Expression Levels

The serum level of sCD163 was positively and significantly associated with the gene-expression level of mRNA CD163 (*r*: 0.37, *P* < 0.001) (Table 3). The level of sCD163 was also found to be significantly associated with the gene-expression levels of IL-10, CD206, and CD68 (*P* < 0.001) and with CD14, TNF-*α*, and MCP-1 (*P* < 0.05). The serum levels of IL-6 and MCP-1 were also significantly associated with all the macrophage markers (*P* < 0.05) (Table 3).

### 3.4. Macrophage Markers and Insulin Resistance

**Figure 3:**
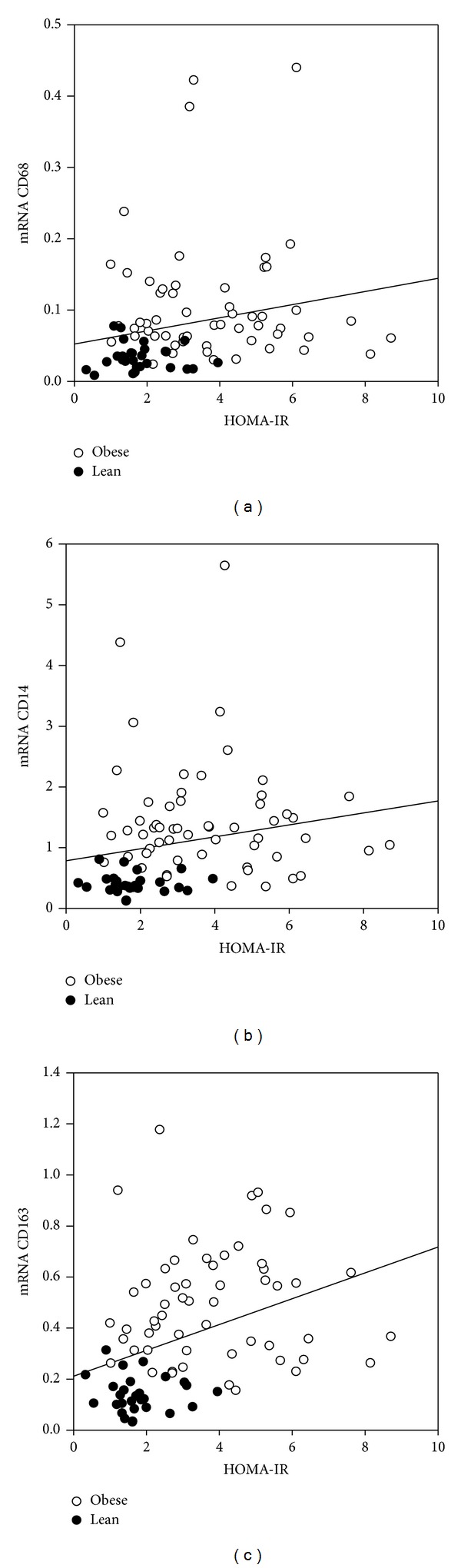
Association between the expression of CD68, CD14, and CD163 and HOMA-IR. Subcutaneous AT samples from lean and obese subjects (*n* = 92). Gene-expression levels of mRNA CD68, CD14, and CD163 measured by RT-PCR. HOMA-IR measured by fasting blood samples. Spearman's correlation with *r* = correlation coefficient. (a) Association between mRNA CD68 and HOMA-IR (*r*: 0.34, *P* < 0.05), (b) association between mRNA CD14 and HOMA-IR (*r*: 0.37, *P* < 0.001), and (c) association between mRNA CD163 and HOMA-IR (*r*: 0.47, *P* < 0.001).

In an univariate correlation analysis a positive and significant association was found between CD68 and HOMA-IR (*r*: 0.34, *P* < 0.05) and CD14 and HOMA-IR (*r*: 0.37, *P* < 0.001) (Figures 3(a)-3(b)). The anti-inflammatory markers, CD163 (*r*: 0.47) (Figure 3(c)), CD206 (*r*: 0.37), and IL-10 (*r*: 0.40), were also found to be positively and significantly associated with HOMA-IR (all *P* < 0.001), and similar association between HOMA-IR and the proinflammatory markers TNF-*α* (*r*: 0.24), MCP-1 (*r*: 0.28), and IL-6 (*r*: 0.27) was found (for all, *P* < 0.05). In a multivariate linear regression analysis CD163 was the only macrophage marker that remained significantly associated with HOMA-IR, also after adjusting the total number of macrophages by CD68 (*β*: 0.57, *P* < 0.05) (Table 4). When adjusting the total number of macrophages by CD14 there was also a positive association between HOMA-IR and CD163 though not significant (*β*: 0.55, *P*: 0.06). The other macrophage markers were not associated with HOMA-IR (*P* > 0.05).

## 4. Discussion

We found that the commonly used macrophage markers CD14 and CD68 were elevated in the AT from obese subjects compared with lean subjects indicating an increased number of macrophages in AT from obese subjects, which is in accordance with several other studies [2, 33]. In a study by Harman-Boehm et al. it is shown that the immunohistochemistry staining for CD68 positive cells highly correlates with the adipose tissue abundance of CD68 mRNA measured by real time PCR [34]. Thus, using gene expression of CD68 (and CD14) may be an acceptable correlate to the number of macrophages.

The gene-expression levels of both the anti- and the proinflammatory macrophage markers were elevated in the AT from the obese subjects compared with the lean subjects. Coherently, we found increased serum levels of the macrophage specific sCD163 and the proinflammatory cytokines IL-6 and MCP-1 in obese subjects compared with lean subjects. This correlates well with the idea that obesity induces a local and systemic low grade inflammation with increased level of macrophages in the AT and increased cytokine production [4]. The serum levels of sCD163, IL-6, and MCP-1 were significantly associated with the gene-expression levels of the ATM markers; however, other cells than the ATMs may also produce and release these cytokines.

To determine if obesity induces a shift in the polarization of the ATMs we measured the ratio of the pro- and anti-inflammatory markers relative to CD14. By this method we adjusted the total number of macrophages in the AT. We found a relatively higher expression of the anti-inflammatory markers, CD163 and IL-10, and a relative reduction of the proinflammatory markers, TNF-*α* and IL-6, in AT from obese subjects compared to lean individuals. Thus, we found that human ATMs change polarization to a more anti-inflammatory profile in obesity than towards a proinflammatory profile, which previously generally had been found both in rodent and human studies [13, 15]. Similar findings were, however, made in a study by Spencer et al. where they compared subcutaneous abdominal AT from lean and obese subjects and found that there was a shift towards a M2 phenotype in non-CLS macrophages in AT from obese subjects compared with lean ones [7]. Furthermore, they found that macrophages in AT from lean subjects expressed a mixed M1-M2 phenotype. A murine study by Shaul et al. also showed an enhanced M2 phenotype in epididymal ATMs from obese mice after 12 weeks of high fat diet compared with mice at 8 weeks [35]. The shift towards a more anti-inflammatory cell type in ATMs from obese subjects may be a protective mechanism to counteract the increased inflammation in the AT seen in obesity. It should be emphasized that both the anti- and proinflammatory markers were enhanced in association with the increased number of macrophages in AT from obese subjects and, therefore, even though there is a shift to a more anti-inflammatory profile, the AT from obese subjects is still more inflamed than the AT from lean subjects. Obesity leads to adipocyte hypertrophy, local hypoxia, and dead adipocytes. Thus, another explanation for the phenotypic switch seen in the ATMs from a proinflammatory state to a more anti-inflammatory state may be due to a need of adipose tissue repair and matrix remodeling.

In the present study we demonstrated that the anti-inflammatory macrophage marker CD163 is highly expressed in the subcutaneous AT from obese subjects and that the gene-expression level is strongly and significantly associated with both CD14 and CD68 (*P* < 0.001). An increased gene-expression level of CD163 in AT from obese subjects compared with lean subjects has been described previously [36]. However, another study found no significant change in CD163+ counts in subcutaneous AT by immunohistochemistry when comparing lean with obese subjects [15]. Additionally, we also found a positive and significant association between the gene-expression level of CD163 and the serum level of sCD163 (*r*: 0.37, *P* < 0.001). Furthermore, the gene-expression level of CD163 was found to be significantly associated with HOMA-IR (*r*: 0.47, *P* < 0.001). A multivariate analysis emphasized this since a positive association persisted between CD163 and HOMA-IR after adjusting for the total number of macrophages by mRNA CD68 (*β*: 0.57, *P* < 0.05). In a study by Wentworth et al. it was found that proinflammatory macrophages were significantly associated with HOMA-IR [17]. We also found a positive association between HOMA-IR and the proinflammatory markers: TNF-*α*, MCP-1, and IL-6, in a univariate correlation analysis (all *P* < 0.05), but in a multivariate regression analysis CD163 was the only macrophage marker that remained significantly associated with HOMA-IR (Table 3). These findings are consistent with our recent data showing a strong association between the soluble part of CD163 (sCD163) and HOMA-IR [23] and consistent with yet another study where soluble CD163 is shown to be an independent predictor of the development of type 2 diabetes [37]. Our results indicate that CD163, both in terms of the gene expression and the soluble part of the receptor, may be of importance in regard to insulin resistance. The background for the link between CD163 and insulin resistance is yet to be understood despite a clear association.

Our study has some limitations. First, only gene expressions are investigated in the AT and gene expressions may not always reflect the protein level. Thus, it would have been interesting to reinforce our results with, for example, immunohistochemistry. Furthermore, we only examined AT from the subcutaneous depot. It would have been of great interest also to investigate visceral adipose tissue. A recent study by Michaud et al. found no depot difference in CD68 mRNA abundance between subcutaneous and visceral AT in lean, overweight, and obese subjects [33]. However, a significant difference in the mRNA CD68 expression between the visceral and subcutaneous depot with elevated levels in the visceral AT has been described [34]. These differences should be clarified in future studies. In the present study we found no age or gender specific difference in the gene-expression levels of CD68 or CD14. However, an association between the macrophage number, measured by the expression of CD68, and age has previously been observed [3, 38]. Thus, the range in age (18–49 years) may be too narrow to detect such an association in our study. Finally, another limitation is that we only analyzed AT from Caucasian subjects and not from other ethnical groups.

In conclusion we found that there is an elevated number of macrophages in the subcutaneous abdominal AT in obese compared with lean subjects. The gene-expression levels of both the anti- and proinflammatory macrophage markers were elevated in AT from obese subjects compared with lean subjects. Furthermore, the serum level of sCD163, IL-6, and MCP-1 was significantly elevated in obese subjects compared with lean subjects reflecting increased systemic low grade inflammation. Unexpectedly, we found a change in the macrophage phenotype by obesity, with a preponderance of anti-inflammatory (M2) markers and a decrement of proinflammatory (M1) markers in ATMs from obese compared with lean subjects after adjusting the total number of macrophages by CD14. A shift in the polarization towards a M2 profile may be a protective mechanism counteracting the enhanced inflammation seen in the AT in association with obesity. Finally, we demonstrated that mRNA CD163 is positively and significantly associated with HOMA-IR also after adjusting for the total number of macrophages and other macrophage markers.

## Figures and Tables

**Table 1:** Primer pairs used for mRNA determination.

	Sense primer	Antisense primer
CD68	5′-GCTACATGGCGGTGGAGTACAA-3′	5′-ATGATGAGAGGCAGCAAGATGG-3′
CD14	5′-AGCCAAGGCAGTTTGAGTCC-3′	5′-TAAAGGACTGCCAGCCAAGC-3′
CD163	5′-CGG CTG CCT CCA CCT CTA AGT-3′	5′-ATG AAG ATG CTG GCG TGA CA-3′
CD206	5′-TTC GGA CAC CCA TCG GAA TTT-3′	5′-CAC AAG CGC TGC GTG GAT-3′
IL-10	5′-AGG GAA GAA ATC GAT GAC AGC-3′	5′-TCA AGG CGC ATG TGA ACT C-3′
IL-6	5′-TTTTGTACTCATCTGCACAGC-3′	5′-GGATTCAATGAGGAGACTTGC-3′
MCP-1	5′-GTCTTGAAGATCACAGCTTCTTTGG-3′	5′-AGCCAGATGCAATCAATGCC-3′
TNF-*α*	5′-TTGAGGGTTTGCTACAACATGGG-3′	5′-GCTGCACTTTGGAGTGATCG-3′
LRP10	5′-AGGTTGCCCAGCACTGAGTTATC-3′	5′-TGCCATCCCACCTGTAGAAGAC-3′

**Table 2:** Characteristics of lean and obese subjects.

	Lean	Obese
Number, *n*	35	57
Age, year	36.7 ± 10.3	37.4 ± 7.5
Gender, female %	51.4	49.1
BMI, kg/m^2^	23.2 ± 1.8	35.6 ± 3.8**
HOMA-IR	1.8 ± 0.8	3.8 ± 1.8**
sCD163, mg/L	1.6 ± 0.4	2.2 ± 0.8**
IL-6, pg/mL	2.0 ± 1.4	2.9 ± 1.6*
MCP-1, pg/mL	90.9 ± 62.8	196.3 ± 102.4**

Data are given for the lean and obese group. Data are mean ± SD or relative frequency (%). Comparison of lean and obese subjects by unpaired *t*-test or Wilcoxon Mann-Whitney rank sum test were appropriated. *P < 0.05; **P < 0.001.

**Table 3:** Association between serum protein levels and the gene-expression levels of the macrophage markers.

	sCD163	IL-6	MCP-1
mRNA			
CD14	0.33*	0.23*	0.50**
CD68	0.38**	0.30*	0.34*
CD163	0.37**	0.34*	0.43**
CD206	0.38**	0.28*	0.47**
IL-10	0.37**	0.23*	0.45**
TNF-*α*	0.22*	0.27*	0.33*
IL-6	0.17	0.25*	0.44*
MCP-1	0.30*	0.28*	0.46**

Subcutaneous AT and blood samples from obese and lean subjects (*n* = 92).

Gene-expression levels of the general macrophage markers: CD14 and CD68, the anti-inflammatory markers: CD163, CD206, and IL-10, and the proinflammatory markers: TNF-*α*, IL-6 and MCP-1 relative to the house-keeping gene LRP10 measured by RT-PCR. Circulating levels of sCD163, IL-6, and MCP-1 measured by ELISA.

Statistic tests: Spearman's correlation test; *r*: correlations coefficient.

*P < 0.05; **P < 0.001.

**Table 4:** Multivariate linear regression for HOMA-IR and M1- and M2-macrophage markers.

	Model 1		Model 2	
	*β*	*P* value	*β*	*P* value
CD68	**—**	**—**	0.01	0.98
CD163	0.57	0.04*	0.57	0.05*
CD206	−0.03	0.89	0.06	0.89
IL-10	−0.47	0.07	−0.07	0.07
TNF-*α*	0.06	0.71	0.13	0.71
IL-6	−0.06	0.47	−0.03	0.49
MCP-1	0.13	0.79	−0.47	0.80

Subcutaneous AT samples from obese subjects (*n* = 57). Gene-expression levels of mRNA CD68, CD163, CD206, IL-10, TNF-*α*, MCP-1, and IL-6 measured by RT-PCR. HOMA-IR measured by fasting blood samples. Multivariate linear regression analysis on a log scale. Dependent variable: HOMA-IR. *P < 0.05.

Model 1 includes all pro- and anti-inflammatory macrophage markers. Model 2 includes all pro- and anti-inflammatory macrophage markers adjusted to total macrophage number by mRNA CD68.
